# The use of non-wood forest products by migrants in a new settlement: experiences of a Visayan community in Palawan, Philippines

**DOI:** 10.1186/1746-4269-2-36

**Published:** 2006-09-07

**Authors:** Celeste Lacuna-Richman

**Affiliations:** 1 Faculty of Forestry, University of Joensuu, P. O. Box 111, Joensuu- 80101, Finland

## Abstract

Migrants are often constrained by a lack of knowledge regarding their new environment and require new skills for their livelihood. In Palawan, some of these necessary skills and knowledge are related to the collection and use of non-wood forest products (NWFPs), many of which the migrants were previously not familiar with. The predominantly Visayan migrants have been successful in familiarizing themselves with the NWFPs in the surrounding forests, with assistance from some of the local indigenous people, in this case the Tagbanua, and from previous migrant settlers. The NWFPs they know about and the extent of use are presented. Currently, except for almaciga (*Agathis philippinensis *Warb.) resin and house-building materials, NWFPs are considered as supplements to agricultural products, not as main source of either subsistence or income.

## Background

Palawan province, with a total area of almost 1.5 million square hectares, has historically been one of the least populated islands in the Philippine archipelago due to endemic malaria and its historical function as the site of a leper community and a penal colony [1]. By the early Twentieth Century however, Palawan became known as a "frontier," where burgeoning populations from other Philippine islands could form settlements and where the chance to own land was great. The end of the Second World War coincided with the government resettlement of large numbers of people into Palawan [2]. Studies of migration into Palawan have emphasized various aspects of the migrant experience, including the sheer difficulty of carving agricultural land from forest and the hunger that comes before the first harvest [3]. Other aspects of the migration into Palawan that have been examined include the role of kinship ties in easing the migration process and the diversification from subsistence agriculture to other income-generating work by second-generation settlers [4,5].

Current attention to communities living in Palawan's forests tends to concentrate on the island's indigenous people, which is necessary, as they have been marginalized by the larger Philippine society for decades. However, the migrants to Palawan are also a vital part both of its past and its future development. The Human Development Report 2004 has discerned some trends in economic and political data regarding the movement of people around the world, both across national borders and in-country. Among them is the rejection of cultural determinism as a measure of economic performance and democracy, intuitively appealing as such beliefs may be. Another trend is the increased political interest in "core values and traits" of people and culture, occurring just as anthropologists have rejected the idea of culture as a restricted and static social phenomenon [6]. The in-country migration of Visayan families to Palawan, and their successful adaptation of forest species to supplement their agricultural produce are indicative of these trends.

There are many reasons why people decide to leave their home and live elsewhere, some having to do with factors within the place of origin, others with perceived possibilities available from the new settlement [7,8]. Whatever the reason for migration, the migrants experience some difficulties and opportunities due to their displacement to a new settlement that those who stay behind may not experience. The difficulties could include doing without the kinship ties that provide some social support and assistance as well as a lack of knowledge about the natural environment. Opportunities, especially for rural migrants can come in the form of agricultural land and relatively abundant forest resources for exploitation [9-11]. However, neither do the difficulties come without relief, nor the opportunities without limitations. In Veloro's study of migrants from the island of Negros to Palawan, relief came in the form of previous settlers offering work and help in familiarizing the new migrants to plants useful for subsistence [3,9]. Hafner and Apichatvullop's analysis of forest land management programs in Northeast Thailand illustrates that migrants can and often do interpret laws regarding the use of land and the harvest of forest resources in their favor, to the detriment of government efforts to preserve the forest. Almost two-thirds of all the household needs of the two predominantly migrant communities in their study, came from the forest. This situation is contrasted with other households in the area who have successfully availed themselves of land-use rights previously and are now agriculturally oriented [11]. In this and other studies non-wood forest products are collected for the household economy either as an important supplement to agricultural production or as the main income source, often resulting in resource depletion [12-14]. Increasingly too, the most important factor in the knowledge and use of "biocultural" resources such as NWFPs may not be the migrant status of the users, either in knowledge lost or gained, but rather the effect of the market economy on the people using these resources [15-17].

The objectives of this study are to find what local NWFPs migrant households use, and how much they use these goods. It is part of a larger study to determine NWFP familiarity and use by various settlement groups in the area, which is characterized by a strong dependence on forest resources and a wide mix of resident ethnic groups, using correlational analysis [18]. The larger inquiry also includes several case studies on practices regarding NWFPs of the indigenous Tagbanua, and this present descriptive study on the NWFP use of the migrant community closely situated to them [19].

### The research setting

Like most villages in Palawan, the population of Dumanguena is a reflection of the larger Philippine population – a mixture of different ethnic groups speaking various dialects, but of basically Austronesian origin. Dumanguena was established as part of Narra, a municipality in which the name is not, as many suppose, inspired by the Philippine National Tree of the same name in the Filipino language (*Pterocarpus indica*), but is an acronym for "National Rehabilitation and Reclamation Area." It was formed by dividing the previously larger Municipality of Aborlan, and is in essence, a municipality chartered to help alleviate the huge population pressures in other parts of the Philippines.

Among the migrants to Narra Municipality, people from the major islands of Negros, Iloilo, Samar, and Leyte, collectively known as the Visayas, are highly represented. Dumanguena's population in particular, is composed predominantly of Visayans, with a few indigenous people living on the margins. Although most of these migrants come from agricultural backgrounds, settlement in Palawan required new skills. The challenge came partly from converting the forests into farmland and partly from having to adjust to the local population. Unlike the implications of the widely-claimed "Last Frontier" status of Palawan, the migrants to Narra did not come to a land devoid of people. The Tagbanua, an indigenous people of Palawan, were already living in various parts of the then Aborlan Municipality. Depending on where within the area they have settled, the Tagbanua lived either by fishing; or by *kaingin *(slash-and-burn) farming, and forest product collection. The Visayan immigrants had to learn to live with the Tagbanua, in some capacity as neighbors, and to some extent because they needed the help of the Tagbanua in adapting to their new environment.

### Study area

Dumanguena Village is located about 89 kilometers south of Palawan's Capital of Puerto Princesa. The village entrance is approximately 11 kilometers to Palawan's western mountains from the National Highway, which runs most of Palawan's length. Politically, it belongs to the Municipality of Narra, although it is closer to the border of Aborlan Municipality than the town center of Narra. The area surrounding the village is agricultural land, planted primarily with rice. Irrigation of the rice fields as well as some of the village's water supply is provided by the Manaile River through a series of government constructed irrigation canals and a dam. The Department of Environment and Natural Resources (DENR) characterizes the soil in the area as sandy clay loam. The mountain range that borders the village and serves as the main collecting site for non-wood forest products is covered with partially degraded forest. This study was based in Manaile, composed of two hamlets within Dumanguena located farthest from the National Highway and closest to the forest. During the selection of study sites, preliminary interviews with the village officials of Dumanguena identified the households in Manaile as the most dependent on the upland forest, as most of the lowland areas have been earlier claimed by previous migrants for agriculture.

The weather bureau of the Aborlan municipality characterizes the climate within the study area as falling within two types. The first type has two distinct seasons, in which the year is equally divided between a six-month wet season and a six-month dry season. The second type of climate is described as having a three- to four- month dry season, and an extended wet season for the rest of the year. During the years 1997 – 1998, the period this study was conducted, the second type of climate could be considered the prevailing type. Respondents to the survey however, have described the years 1997 and 1998 as a period of "extremely" irregular weather. The period of "El Nino", in late 1997 to early 1998 was considered very dry and conducive to forest fires. The latter part of 1998 and early 1999, in contrast, was regarded as a period of almost constant rain. The climatic conditions reportedly had adverse effects on the cropping patterns and other seasonal work of most of the respondents. As far as NWFP collection was concerned, the climatic effects of El Nino was mixed. The respondents reported larger amounts of resin from almaciga (*Agathis philippinensis*) trees, which thrive under dry conditions, although there was a fear of forest fires destroying the stands. Herbacious NWFPs used for food, fiber and medicine, on the other hand were not as easily available as during non-El Nino years.

## Materials and methods

The data for this study was collected by face-to-face interviews using semi-structured and open-ended interview schedules. There was an attempt to interview all the heads of households in Manaile. However, due to refusals, only 45 individuals who were the acknowledged heads of their households were interviewed as part of the main survey, which comprised approximately 56 percent of all the households in Manaile. More than half (57%) of the respondents were female, all except one had formal schooling, and although the ages of the respondents ranged widely from 20 – 78 year old, all of them were the ones in charge of their respective household's finances. Heads of households who refused to be interviewed usually gave one of two reasons for their refusal. The first was the lack of solid results from previous government surveys, and the second was the fear that the results of such surveys would be used against them in the future. Convincing these heads of households otherwise was unfortunately not successful. During the interviews with the official respondents (head of household), input from other family members was also recorded. Interviews with key informants outside the village, and participant observation, which involved attending village meetings with the permission of the residents, were also done to support or clarify information supplied by the survey interviews.

The first phase of interviews were done during the early part of 1998, to establish the familiarity of the respondents with the species used as NWFPs. There were two visits for each respondent. An initial list of NWFPs commonly used by the household was asked from each respondent during the first visit. After compiling these lists into one for the whole community and checking each plant against botanical references, the species listed were confirmed during a second visit. This was done by asking each respondent whether they were referring to the same plant, if it was in their household list. If the plant was correctly identified, the respondent was asked how it was used, and how often it was used in their household.

The second phase of interviews was accomplished in the latter part of 1998 and January-February 1999, to affirm or correct the answers during the initial interviews, and to ask for additional information. Other forms of participant observation, including visits to the forest with NWFP collectors, visits to the local markets every week, and to dealers of selected non-wood forest products occurred also throughout 1998–1999 [18-20].

## Results

### Socioeconomic profile of Manaile, Dumanguena

Dumanguena is fairly representative of larger Philippine villages. There are services such as primary and secondary schools and a health center. There are several "*sari-sari*" (sundries) stores, which are in and out of business depending on the proprietor's ability to raise capital. Also like many Philippine villages the health center is open irregularly and can only give the most rudimentary of health care when operational; and the stores stock only foodstuffs and a few other items. Most of the villagers use resources directly from their natural environment without the services of a utility company. There is limited and sporadic electrical power supply in some parts of the village for those who can afford the connection fee. Everyone else depends primarily on kerosene and charcoal for their lighting and cooking needs, with the odd private generator used sporadically. Water is accessed from the river, from irrigation ditches, and from a community deep-well with hand pump. Yet, Dumanguena is different from many Philippine villages because of the composition of its population. It has a fairly homogenous population in terms of language and ethnicity, but the origins of the villagers lie elsewhere in the Philippines. In most cases, their ties with the people, culture, and natural features of the areas from which they come is still very strong.

In Manaile especially, the majority of the respondent households (86.4%) were migrants from Provinces of Antique, Panay, Iloilo, Negros, Leyte, Samar, and Cebu, collectively known as the Visayas. The rest of the heads of households interviewed (13.6%) were born in Palawan, the second-generation of Visayan families in their new settlement. Many of the respondents were recent migrants, arriving in Palawan in the late 1980s and 1990s. They had chosen to live in Dumanguena partly because they already knew of some people from their province of origin who have successfully moved to this place.

The success of Visayan migrants to Palawan could be measured greatly by one feature of a family's status, which is land ownership. Most of the migrant families had agricultural backgrounds, but none of them owned any farmland in their province of origin. The lack of land in their respective home provinces was cited by the majority of respondents as the major reason why they moved to Palawan. This reason is supported by a study on community and family factors that affected migration from another Philippine province, showing a significant relation between agricultural assets and migration in communities [7]. Ironically, in Dumanguena, patterns of land ownership are beginning to acquire some features of the provinces from which the migrants come. Although there is no land tenancy as such and the phenomenon of absentee landlords has not yet taken root, there are occasionally rice paddies that were hired out to landless agricultural workers who got paid a percentage of the harvest. The average land holding among the respondents was 0.85 hectare. This includes the home lot, which is uniformly set (by the Department of Agriculture and the municipal government) at 20 × 20 meters per household, and usually a plot for growing rice and vegetables for family consumption. Only two respondents owned more than two hectares of lowland rice paddy.

The average head of a household was 38 years old, and may be male or female. There was separation of duties in most households, but except for the men's resin collection and hunting, these were not strictly followed. Resin collection and hunting were considered male activities mostly because they required working in teams sometimes overnight, and most respondents wanted to work in single-gender teams. Although there was one respondent who had not gone through any formal schooling, the average household head had finished the primary level of public school education in the Philippines, which is up to sixth grade. Many among the respondents had attended a year or more of secondary school and a few (7%) had attained a college degree. Like most Filipinos, the respondents valued education highly not just for personal improvement, but as a source of clan wealth, and as a hedge against family misfortune. In a sense, the collective educational level of the entire village also had some bearing on their utilization of non-wood forest products and their relations with the Tagbanua, who were also NWFP collectors.

There was a mean of three dependent children per household in Manaile. As it is also common in the rest of rural Philippines, single adult offspring continue to live in their parent's home, if they reside in the same area. However, for the most part these adolescent/adult offspring have control over their finances, even as they defer authority for the running of the household to their parents. For this reason, all the respondents who are the head of their respective households are either married or widowed. The living arrangements of people within their households also had bearing on the family income. When asked about the income of the household, the respondents included all sources, especially contributions of non-dependent family members to the household budget. When the adult offspring marry, the majority of parents disoblige them from contributing to the upkeep of the family. The practice of augmenting the family income while single and being excused from the duty once an individual marries, is described in other studies of Philippine rural households as well [8].

Dumanguena is a predominantly agricultural area and is geared towards the production of rice (*Oryza sativa *L.). Thus, the kind and intensity of work is highly seasonal in nature. In a typical production year, the months of January to March are spent to clearing of rice fields, and planting is done in April. The rice-growing season is usually from April to August or September. After the harvest, some families will try for a second cropping of rice. Other crops are considered of secondary importance to the rice crop, and are planted either in home gardens or in *kaingin *farms located in steeper terrain throughout the year.

All the household heads interviewed have both a primary and secondary source of income. Primary income source is defined in this study, and explained to the respondents, as the work that best provides for their family's material needs. The secondary sources of income are whatever augments this work. Among the primary sources of income, subsistence farming is the most widely practiced (38.64%), followed by collection of almaciga (*Agathis philippinensis *Warb.) resin (20.45%) and rice farming (18.2%). Other occupations within the community are performed by specialized individuals, including the teachers in the village school (6.82%), farm laborers (4.55%), a carpenter, store owner, *arbularyo *(traditional healer), and village headman.

The role of non-wood forest products in the livelihood of Manaile residents is seen also as one of their secondary sources of income. Apart from the households who consider almaciga resin collecting as their primary source of income, is an additional sixteen percent of the population who consider resin collecting as their most important secondary income source. Almost half of those who collect resin also gather rattan (*Calamus spp, Daemenorops spp., Korthalsia spp*..), another valuable commercial non-wood forest product.

For a number of skilled men, hunting is a lucrative source of extra income. Wild pig meat is a highly desired commodity and subsistence food. All respondents who hunt were willing to keep their game for the consumption of their immediate and extended family, if they could. Those who had more resources made efforts to keep all the wild pig meat for family use, disregarding the relatively high price they could get for it when sold. However, if they decided to sell some of the meat, there was no danger of lack of buyers despite the low income levels in the community. According to certain hunters, the capture of a wild pig is frequently the only way some of them can get animal protein due to the high cost of meat. Usually, they get 30 to 60 kilos of meat from a female wild pig, and 80 to 100 kilos from a male. The less well-off hunters usually only keep the head and feet of the wild pig and sell everything else. The technique used for hunting wild pig is similar to that of the Tagbanua, who use the "pig bomb", composed of usually rotting fruit with an explosive inside, and not the use of traps, which is the most common method in Leyte, an island in the Visayas group, where wild pig meat is also an important commodity [19,21]. This in itself may provide an indication of the high degree of contact between the migrants and the indigenous people in Palawan, as far as NWFP use is concerned.

The mean household income in Manaile is 29,740 Pesos per year (about 743 USD in 1998). The mode is 36 thousand Pesos (900 USD in 1998), but some respondents' livelihoods are almost completely subsistence-based, and they claim very little income and even less disbursements. The villagers cultivate a variety of crops (n = 68) in their home gardens and *kaingin*. Most of these plants are species they are familiar with from their province of origin, but due perhaps to the greater availability of land in Palawan, they have not been able to grow these until their settlement in Manaile.

All the respondents who engage in agriculture claim that they produce for their respective families, but that the possibility of selling their crops is never far from their minds. The most commonly cultivated plants apart from rice are banana (*Musa sapientum *L.); nangka (*Artocarpus heterophyllus *Lamk); Palawan gabi; mango (*Mangifera indica *L.); avocado (*Persea americana *Miller) and squash (*Cucurbita *L.) [22,23].

### Species used

Unlike the plants cultivated by the villagers, most of the species the villagers utilize from the forests were once unfamiliar to them. The Tagbanua, an indigenous people of Palawan island, or previous settlers from other Philippine provinces, introduced these plants to them. Many respondents admitted to wariness in trying some of the wild fruits and forest vegetables that they have encountered only in Palawan, but said that they now enjoy and value these plants. The introduction of most of these forest plants to the migrants often occurred when they joined the Tagbanua in teams for collecting almaciga (*Agathis philippinensis*) resin, and from sharing such plants with other households on their return from the forest [19,20]. Evidence of their acceptance of these plants is shown in Table 1, which contains a list of NWFPs used by the respondents' households and the frequencies with which households utilize these. The respondents were able to recognize most of the NWFPs from the list compiled from the initial interviews. Although the percentage of plants used among those known is quite high (78%), only a few of these NWFPs were used fairly frequently. Of the NWFPs used, only 2% were used on a daily basis, 16% were used more often than once a day, 5% were used weekly, and 12% monthly. Most households used NWFPs on a yearly basis only (37%). A common reason for the yearly or infrequent use of some species is their availability. The respondents claimed that they use some NWFPs on a yearly basis (Table 1), mostly because these species are only available seasonally. Most collect subsistence NWFPs opportunistically, i.e., when going to the forest to collect resin or bamboo, commercial NWFPs, on planned trips. Almost all respondents said they would use more NWFPs if they were more readily available (grow abundantly throughout the year), and more easily collected without the need to go further in remote steep mountain areas of the forest.

**Table 1:** Non-wood forest products used in Manaile, Dumanguena, Palawan

		Frequency of use (no. of households)				Uses		
Scientific Name/Family	Visayan Common name	Daily	Weekly	Monthly	Yearly	Food	Medicine	Fiber
ANACARDIACEAE								
*Dracontomelum edule *(Blanco) Skeels (from Phil. Her. vol.1) – alauihau	halowihaw/alauihau	0	0	0	21	x		
*Mangifera altissima *Blanco	pao/paho	0	0	1	10	x	x	
*Mangifera indica *L.	Mangga	1	1	1	16	x	x	
*Mangifera odorata *Griffith	wani/huani	0	0	0	0	x	x	
*Semecarpus longifolius *Blume or *Semecarpus gigantifolia *Vidal	anagas/kasoy-kasoy	0	0	1	10	x		
								
ARACEAE								
*Colocasia esculenta *(L.) Schott	gabi/dagmay	2	4	9	6	x		
								
ARAUCARIACEAE								
*Agathis philippinensis *Warb./*Agathis dammara *(Lamb.) L. C. Rich.	almaciga/sahing/salung	2	1	10	10			
								
ARECACEAE								
Oncosperma tigillaria Ridl.	Anibong	0	2	3	11	x		x
Orania decipiens Becc.	Banga	0	0	1	10	x		x
*Arenga ambong *Becc. or *Arenga brevipes	batbat	0	5	7	9	x		x
								
BOMBACEAEAE								
*Durio zibethinus *Murray	wild durian	0	0	2	16	x		
								
BORGINACEAE								
Ehretia acuminate	Anonang	0	2	7	9	x		
								
CONVOLVULACEAE								
*Ipomoea batatas *(L.) Lamk.	kamoteng bagin	0	4	11	5	x		
								
CRASSULACEAE								
*Kalanchoe pinnata *(Lmk) Pers.	anheliko/anhelika	1	5	7	3		x	
								
CYCADACEAE								
*Cycas circinalis *L.	petogo (sago)	0	0	5	3	x		
								
DIOSCOREACEAE								
*Dioscorea hispida *Dennst.	kayos (kuyot?)	1	0	1	11	x		
*Dioscorea pentaphylla *L.	Sapang	0	0	3	23	x		
								
DRYOPTERIDACEAE								
*Athyrium esculentum *Copeland	Pako	0	0	2	9	x		
								
EUPHORBIACEAE								
*Mallotus auriculatus *Merr.	Kamanian							
*Mallotus pseudopenangenensis*	beri/beli	0	0	1	9	x		
								
GNETICEAE								
*Gnetum gnemon *L. Var. gnemon	Bago	0	4	11	9	x		
								
FLACOURTIACEAE								
*Caesaria fuliginosa *(Blanco) Blanco	luyong-luyong	2	4	5	11	x		
								
GRAMINEAE								
	tapikan (bamboo)	0	1	3	9	x		x
*Bambusa *sp., *Schizostachyum *spp.	Kawayan	0	1	11	8	x		x
								
GUTTIFERAE								
*Garcinia benthami *Pierre or *Garcinia dulcis *(Roxb.)Kurz.	Bunog	0	1	2	11	x		
*Garcinia binucao *(Blanco) Choisy or *Garcinia lateriflora *Blume	kandis/kindis	0	1	3	23	x	x	
								
LAMIACEAE								
Mentha cordifolia Opiz.	herba Buena	3	4	6	3		x	
								
LEGUMINOCEAE								
*Cajanus indicus *Sprengel	Kadyos	2	0	2	19	x		
								
MENISPERMACEAE								
*Arcangelisia flava *(L.) Merr.	albatra/albutra	0	0	6	2		x	
*Cocculus cordifolius*-medicinal boost to autoimmune system	Makabuhay	1	1	6	7		x	
								
MORACEAE								
*Artocarpus heterophyllus *Lamk.	nangka/langka	1	2	5	14	x		
*Litsea perrottetii *(Blume) F. – Vill (Laur.) or *Artocarpus odoratissimus *Blanco	Marang	0	1	1	7	x		
								
MYRTACEAE								
*Eugenia cymosa*	labak/beraho	0	1	0	4		x	
*Syzygium cumini *(L.) Skeels	duhat/lumbay/lomboy	0	2	1	17	x		
								
MUSACEAE								
*Musa *L.	Saging	6	7	3	4	x		
								
ORCHIDACEAE								
	Orchids	0	1	5	12			
								
OXALIDACEAE								
*Averrhoa bilimbi *L.	Kamias	0	5	4	9	x		
								
PALMAE								
*Calamus *sp.	yantok/rattan	0	3	13	8	x		x
*Calamus ornatus *Blume	Alimuran	0	2	7	3	x		
								
SAPINDACEAE								
*Nephelium lappaceum *L.	manti (Tagbanua name)	0	0	3	28	x		
*Nephelium lappaceum *L.	Rambutan	0	1	1	24	x		
*Guioa acuminata *Radlk.	Pasi	0	0	1	25	x		
								
SAPOTACEAE								
*Pouteria campechiana *(Kunth) Baehni	Tisa	0	1	2	19	x		
								
SCHIZAEACEAE								
*Lygodium circinnatum *(Burm.) Swartz	Nito	0	1	3	10	x	x	x
								
STILAGINACEAE								
*Antidesma bunius *(L.) Sprengel	Bignay	0	1	0	11	x		
								
TILLIACEAE								
*Muntingia calabura *L.	Seresas	1	3	5	8	x		
								
Others:								
								
*Dimonorops curranii*	pitpit/pitpiton	0	1	1	5	x		
*Emilia marivelensis*	libhon/libun	3	8	6	2		x	
*Lentinus *sp.	kulat/alasan	0	2	1	2	x		
*Melodinus luzonensis*	Tabo	0	0	3	27	x		
	Balisangkad	0	0	0	17	x		
	Banga	0	0	1	10	x		
	Palawan gabi	1	3	3	13	x		
	Karngangyan	0	1	4	7	x		
	Kulumbi	0	0	5	13	x		
	laba/balokawe	0	2	6	9	x		
	Lampanaya	2	6	9	1	x		
	Lipso	0	0	0	18	x		
	pakpak lawin	2	2	1	9	x		
	Pal	0	1	0	1	x		
	Palawan durian	0	0	1	14	x		
	sangi/pangi	0	0	0	8	x		
	santol-santol	0	1	1	12	x		
	Tagumo	1	0	4	2	x		
	Tuge	0	0	2	10	x		
	wild gabi	1	1	1	6	x		

### Forest food

In general, the villagers depend on their own crops for the food they eat, supplemented by fish purchased from traders who bring it in from communities located near the coast, and fish caught in the river. However, there is a consensus that if a monetary value will be assigned to the food they consume, the income that they earn will not be sufficient. The respondents were asked how they augment their food budget. A large percentage of the respondents practice the combined strategy of growing their own food and collecting edible forest products (34%). In addition to this group are those who exclusively depend on forest food collection to augment their dietary needs (7%), and those who exclusively depend on their own crops for food (23%). Various other strategies include working for food (7%); borrowing money (4.6%); and in about equal measure: depending on relatives for their subsistence, hunting, and combinations of the different strategies.

While there is considerable use of edible forest plants, this does not always represent a "free" good for the households. There is an informal trade of edible non-wood forest products that exist between the Visayan villagers, and the Tagbanua. Members of the settlement who have migrated to the area earlier remember other forest food as survival food, particularly the pith of certain rattans and bamboo shoots. These were necessary until the rice fields and homegardens were well-established and productive. Presently however, these foods have become standard items in the diet of the villagers. Some of these forest foods have made the transition from being considered hardship food to delicacies fit for celebrations. An example of this kind of forest food is the pith of the *batbat *(*Arenga ambong *Becc.), which when cooked with meat, is often served at weddings and other fiestas.

Two groups within the villagers were particularly high consumers of forest food, both in terms of species identified, and the amounts of each specie harvested. These were the households who had members, mostly male, who collected almaciga resin; and the households whose *kaingin*, or agricultural plots cleared from forest, were accessible only by passing through upland forest. For these two groups, which often overlap, the opportunistic collection of forest food was more frequent than for those households that had no commercial NWFP collectors, and for which their *kaingin *was closer to the plains. Forest plants often serve as emergency food when their stores of rice run out during week-long stays in the forest. Members of households who have almaciga collectors are also quite likely to collect enough forest food both for their own consumption and for sale. One respondent has attempted selling *kandis *(*Garcinia binucao *(Blanco) Choisy) a wild fruit which is not usually sold in Palawan, but is sold in Antique, their home province in the Visayas. The *kandis *fruit is a berry 3 to 4 cm. in diameter, with numerous seeds, and very acid. *Kandis *grows at the forest edge, and is thus relatively easy to harvest when fruits are available. Other respondents have attempted to cultivate this plant, but although some vegetative growth has been successful, it is difficult to predict when the fruiting season occurs. For these reasons, the migrants who have attempted cultivating *kandis *as a backyard crop in Palawan have not been encouraged in their endeavor.

### Medicinal plants

The use of forest plants for medicinal purposes was considered both an immediate and alternative solution to curing illness. Although a plurality (32%) of the respondents only depended on doctors and the municipal hospital in Narra, more than a fifth (23%) of the respondents consulted a licensed medical doctor and an *arbularyo *or traditional healer, equally. Eighteen percent went to the village health center run by volunteers and occasionally to a registered nurse for their medical needs. An additional nine percent of the respondents went to the village health center and the hospital in Narra. Apart from the five percent of the respondents who practiced self-medication using forest plants and traditional healing methods, the rest (13%) used various combinations of the above-mentioned ways of curing themselves, depending on the perceived severity of the illness and their financial standing at that moment. As a rule, those who had the means to go to a licensed medical doctor or hospital did so.

The respondents were asked about their use of medicinal plants, regardless of their preference for medical assistance. Forty-five percent (45%) of the respondents stated that "herbals" or medicines concocted from both wild and cultivated plants were an essential part of their families' health care. Initially, some of the users of these plants asked help from the *arbularyo *to learn about the plant, the method of preparation, and the right dosage. Methods of preparation are frequently based on the nature of the ailment, e.g. decoctions were made for internal problems, poultices for wounds, aches and pains, or on the nature of the plant part, e.g. powders from tree bark or roots. The medicinal plants used include locally well-known items, such as leaves of the guava tree (*Psidium guavaja*) as poultice for wounds, but there were also plants to which various medicinal properties were prescribed by arbularyo. An example of the latter is *makabuhay*, or *panyawan *(*Tinospora crispa *(L.), which had also been used for healing wounds, decocted for stomach aches, and like one or two other plants, used as an anti-malarial drug by settlers in an area where malaria is a common and frequent concern. Eventually, the majority of medicinal plant users prepared and administered the dosages themselves, for members of their household. However, all the users of medicinal plants admitted that if commercially prepared medicine were more affordable, they would probably have used these instead.

### Housing

The role of non-wood forest products in the livelihood of the villagers and the relevance of ensuring their sustainability was most apparent in their houses. All the respondents consider Dumanguena a permanent settlement, but they characterize their houses as native, referring to the materials used, which were temporary, but readily available for future repairs. These houses were made almost exclusively of non-wood forest products, particularly bamboo (*Bambusa spp, Schizostachyum spp*.), nipa (*Nypa fruticans *Wurmb.), anibong (*Oncosperma gracilipes *Becc.) and rattan [24].

Of the respondents' homes, only one house was made of concrete and lumber (2.3%). The rest of the houses in Manaile were either made completely of NWFPs (79.6%), or built partly with NWFPs and partly with other materials such as lumber or metal sheets (11.4%). The remainder of the respondents did not own the houses in which they resided (6.8%), but the houses they rented, were also made in part with NWFPs. All the respondents who owned the houses they lived in claimed that they had either gathered all, or most of the materials used to build their homes themselves, although the house-building itself required some paid labor. Most respondents did not have the opportunity to buy building materials, therefore had little choice but to gather NWFPs.

The respondents' estimates of the monetary costs for construction of a typical family dwelling in Manaile were based mostly on how much was paid for labor, which varied widely depending on the kinship and good relations between the prospective home owner and the laborers. However, the value of the materials could be estimated from the existing prices for NWFPs for sale at markets in the area. Nipa shingles cost 170 Pesos per 100 pieces, and a house in Manaile would probably require about 700 pieces. A 2.5 × 8-meter *sawali *panel which is made of woven bamboo strips, cost 180 Pesos at the "forest gate," and approximately ten to fifteen such panels would be needed. Bamboo or *anibong *poles for flooring cost 35 Pesos per bundle of ten. The typical village house with a floor space of approximately 30 to 50 square meters could easily cost the owners almost a year's average earnings, if they had bought the materials instead of collecting them themselves.

### Trade in Non-wood Forest Products

The most common commercial NWFPs were rattan and resin (exudate) from almaciga trees, both of which have been in danger of depletion as early as the turn-of-the-twentieth century [13]. The Visayans practice the same extraction methods and collected similar amounts of these NWFPs as the Tagbanua, although there were many anecdotes concerning how the Tagbanua can carry much heavier loads of resin, up to 60 kilos, from the mountains than the migrants can [20]. However, since a number of villagers also held renewable interim licenses to extract these NWFPs, their percentage of the income from whatever they collected was usually larger than the Tagbanua collectors, even as the Tagbanua have applied for a Certificate of Ancestral Domain Title (CADT) for the forest area for more than a decade [19]. Other collectors ask those who hold licenses to sell their resin for them, and the license holders are also usually the leaders of almaciga resin collecting teams. To some extent, the greater success of the migrants in obtaining licenses or marketing the resin is based on geography. They do not have to cross a river like the Tagbanua, and are located closer to the National Highway than the Tagbanua village. Other reasons for their relative success could also be attributed to higher literacy levels, and more contacts in the provincial capital, Puerto Princesa.

Almost all of the respondents interviewed were interested in collecting flora and fauna from the forests for which they had what may be called an aesthetic rather than a physiological need. A large number of the villagers had ornamental plants which they claimed have no medicinal or edible uses, and kept different kinds of forest animals as pets, notably the *balintong *or spiny anteater, monkeys, and the *kiyaw *or talking mynah bird. However, the respondents were willing to sell these plants and animals should someone offer them a reasonable price for these. There is an existing trade in forest animals in Manila, but it is tacitly assumed by both potential buyers and sellers to be illegal. Thus, the lack of trade in forest animals from Dumanguena is not due to the reluctance of the collectors, but according to them, by the absence of buyers willing to take the risk of bringing such animals to the Manila market.

## Discussion

It is possible that new migrants face certain risks in their new environment that earlier settlers had already learned to manage. Yet, the same migrants may also introduce knowledge and skills from their homeland that could enhance the use of resources in the new settlement. This situation was repeatedly observed in Dumanguena, about the villagers' utilization of NWFPs. In comparison with a similar study of indigenous people living within the same area, it was found that the Visayan migrants identified almost the same number of useful forest plants as the indigenous Tagbanua [18]. There was a keen and obvious interest by the respondents in the potential of cultivation and commercialization of any promising NWFPs. Certain individuals in the community had already attempted to grow some forest fruit trees and almaciga within the village confines, although the results had not been successful.

Despite the lack of success in cultivating promising forest plants to date, there was still a certain optimism regarding the cultivation and marketing of some commercial NWFPs. As mentioned earlier, some of this optimism was due to the migrant villagers' preference for tending crops within their homegardens and other more accessible land which they own, in comparison with having to compete for the collection of such resources in common areas such as the forest. Another reason was that the villagers had heard stories regarding the profit from some plant products in urban markets, and they recognized these plants as being available in their forest area. For example, in 1998 – 1999 there had been great interest in the noni fruit (*Morinda citrifolia *Linn.), the juice of which a foreign company was selling in Manila for approximately 2000 Pesos per liter. The uses of noni fruit in different Polynesian and Southeast Asian islands as food, fodder, fabric dye, and medicine was well documented [25]. However, in the Philippines the excitement about this fruit, locally known as *ampatot*, had to do with its touted ability to cure all kinds of ailments. Whether or not those claims were valid, some villagers in Manaile had enough contacts with relatives in the Provincial Capital of Puerto Princesa, or in Manila, to know that the fruit sold at a very high price. Inspite of this knowledge, any *ampatot *was observed selling in the local markets for only 10 Pesos per kilo. Clearly, the aptness in finding a commercial niche for a forest product, does not easily lead to maximizing the possible returns from such a product.

The case of noni, which was a relatively new forest product in local markets, is mirrored in other NWFPs as well, even those that had been in national and international markets for a few decades and possibly a few centuries. Almaciga resin, for instance, had been recorded as a common item of trade in Palawan between the indigenous people and Chinese traders even before Spanish colonization in the 1600s [26,27]. However, even at present, the final processing of such value-added products from the resin such as lacquer, adhesives, and paint are done in the countries importing the resin.

The lack of local markets, processing facilities, as well as communication and transportation services, prevents the commercialization of certain non-wood forest products from providing greater financial benefit to the gatherers. Most respondents are quite aware of the shortcomings of the system, but did not have the capital or the connections to remedy the situation. The commercialization of specific NWFPs often led to a more intensive free-for-all in harvesting of the products, which lead to depletion. For instance, instead of initiatives to do some intermediate processing of almaciga resin within local communities for greater profit, many almaciga gatherers had individually decided to collect more resin. The conflict over certain trees, and the difficulty of ensuring that every gatherer got a fair share of resin, had been such that a number of individuals had resigned from leading collection teams. As expected, the increasing influx of new migrants will probably make competition for almaciga resin collection more intense, as these new in-migrants will have less land to claim for agriculture. It is a fair guess that the almaciga trade cannot sustain additional numbers of collectors, nor support those who are depending on it at present without new stands being established.

In contrast to the looming scarcity of commercial NWFPs, subsistence goods from the forest such as edible and medicinal plants did not seem to be in any danger of provoking conflict among gatherers, despite the significance of these to the settlers' livelihood. Thus, these are indefinitely sustainable, a situation that may change only if there is expanded transformation of forest land to agriculture, or, if these subsistence NWFPs find a sizeable market. There has been an attempt in this study to quantify how much of the frequently mentioned subsistence NWFPs were collected. However, exact figures could not be quoted, since the gatherers of such subsistence forest products collected only as much as they could use immediately. This amount varied from a handful of forest greens for a meal, to several poles of bamboo for house repair.

Observations on subsistence level gathering of NWFPs by the villagers are supported by the presence of these products in local markets. "*Tabuan*," the once-a-week local markets in rural areas, was the common venue for the sale of any excess NWFPs such as fruits, resin, and construction canes. Frequently, only one or two sellers of NWFPs of any kind were seen at these markets; at most there are five sellers of these products. Those that sell forest fruits, vegetables, resins, and fibers, only had a few of these products for sale, which were minor items, the bulk of what they offered being mainly agricultural products.

Very few of the respondents admitted to selling any NWFPs. Those who said that they had sold NWFPs that were considered subsistence goods only did so rarely, and in small quantities, like two or three pieces of the larger fruits. According to some of these respondents, they only "experimented" with selling these goods, since they were not sure that anyone would buy them. The reasons given for frequent gathering of forest plants in small quantities were the plants' abundance in the forest, and the short shelf life they had.

## Conclusion

The status of NWFPs in the settlement of Dumanguena depends on whether these products were predominantly commercial or subsistence products. Plantations of commercial NWFPs such as almaciga resin and rattan could possibly be beneficial to the gatherers of these products, due to the depletion of these resources in the natural forest. However, the land ownership/stewardship issues that underlie any plans for plantation establishment in Palawan make such plans almost impossible to set in motion. A viable option for almaciga is to replace dead or badly damaged trees in the forest with either planted seedlings, or allowing naturally regenerated almaciga trees to grow before attempting to extract resin from them [20]. Like other commercial NWFPs, the problem with almaciga resin and rattan does not stop at ensuring supplies. Greater supplies of these products would not improve the lives of the villagers greatly unless there was a better system for getting these products to the market. A better system in Dumanguena's case would include more efficient organization for sorting and pricing the raw almaciga resin and rattan cane within the community itself, and a way for gatherers to communicate with main buyers in the Capital, without having to go to Puerto Princesa.

As for subsistence NWFPs such as food and medicine, there does not seem to be any need in the near future for their cultivation or regulation. There was virtually no local market for these products at present, and attempts to grow them in homegardens had not been successful. However, having said this, there was also a great need to preserve the natural forest from which these subsistence products were collected, since the conditions for their growth in places other than the natural forest were still largely undetermined. In the meantime, they provide goods to the villagers, necessities that cannot be bought due to the low income levels in the community. Results of the interview reveal that the only limit to the utilization of subsistence NWFPs was the willingness of the user to collect these products, and in the case of medicinals, the users' belief in their efficacy. The NWFPs used for subsistence purposes either became a permanent part of the diet and material culture of the migrants, or, were simply discarded for agricultural produce and commercially manufactured products when these could be afforded. However, during times when cash was unavailable, which was often, NWFPs were again resorted to. The arrival of new migrants to the area, which is a distinct possibility, also requires that there is some form of livelihood available for them. With little land for further agricultural expansion, it is probable that NWFP extraction will be one of the first sources of income and subsistence for the newcomers. In summary, the loss of NWFPs would have great social and not just environmental implications in Dumanguena, despite the fact that most of the villagers have not been familiar with the resources available in their new settlement from their childhood.
